# Experimental Validation of a Compact and Versatile Bioimpedance Measurement Platform Based on the SENSIPLUS Chip

**DOI:** 10.3390/s26154922

**Published:** 2026-08-04

**Authors:** Lorenzo Giannini, Rita Asquini, Alessio Buzzin, Simone Contardi, Paolo Bruschi, Emanuele Piuzzi

**Affiliations:** 1 Department of Information Engineering, Electronics and Telecommunications, Sapienza University of Rome, 00184 Rome, Italy; lorenzo.giannini@uniroma1.it (L.G.); emanuele.piuzzi@uniroma1.it (E.P.); 2 Interdisciplinary Department of Wellbeing, Health and Environmental Sustainability, Sapienza University of Rome, 02100 Rieti, Italy; alessio.buzzin@uniroma1.it; 3 Sensichips s.r.l., 04011 Aprilia, Italy; simone.contardi@sensichips.com; 4 Department of Information Engineering, University of Pisa, 56100 Pisa, Italy; paolo.bruschi@unipi.it

**Keywords:** bioimpedance, wearable sensors, telemedicine, physiological monitoring, Internet of Medical Things

## Abstract

The growing demand for wearable and Internet of Medical Things (IoMT) devices is driving the development of compact, low-power platforms for continuous physiological monitoring. Bioimpedance analysis represents a versatile non-invasive technique for the assessment of tissue properties, body composition, and respiratory dynamics. This work presents a comprehensive experimental validation of a compact bioimpedance measurement platform based on the SENSIPLUS chip, a CMOS sensor interface integrating a frequency-programmable lock-in amplifier for Electrochemical Impedance Spectroscopy in the 10 kHz–1 MHz range. The platform was validated at three complementary levels: (i) electrical characterization on Debye tissue-equivalent circuits using a three-point bilinear calibration, with analysis of the electrode–skin contribution and repeatability assessment; (ii) in vivo multi-frequency bioimpedance spectroscopy (BIS) with Cole–Cole model fitting and hook-effect correction; and (iii) single-frequency thoracic impedance plethysmography for respiratory monitoring. Results were compared against an Agilent E4980A precision Inductance (L), Capacitance (C), and Resistance (R) meter and a calibrated spirometer. The presented device achieved a maximum resistance error below 5.7% and reactance deviation under 6 Ω across the investigated frequency range, Cole–Cole parameters consistent with reference values, and strong linear correlation ($R^{2}=0.97$) between thoracic impedance variations and tidal volume, with respiratory rate estimation errors below 2% across the ten sessions, specifically 1.43% during normal breathing and 1.96% during deep breathing. These results demonstrate that the SENSIPLUS-based platform achieves metrological performance compatible with the requirements of wearable IoMT applications, here demonstrated in a single-subject proof-of-concept study, while relying for all critical analog functions on a compact (1.5×1.5) mm^2^ system-on-chip with low power consumption (1.5 mW).

## 1. Introduction

Recent advances in telemedicine, personalized healthcare, and Internet of Medical Things (IoMT) technologies are driving the development of compact, low-power, and non-invasive systems for continuous physiological monitoring [1,2,3,4]. In this context, wearable sensing platforms are attracting increasing interest because they can enable remote and longitudinal assessment of patients, reduce the need for hospital-based examinations, and support earlier detection of clinically relevant changes in health status [5,6].

Among the physiological variables of interest, respiratory function and body composition are particularly relevant in the management of chronic conditions. Conventional reference techniques, such as spirometry and polysomnography, remain indispensable in clinical practice, but they are not ideally suited for continuous and unobtrusive monitoring because they require trained personnel, dedicated clinical settings, and, in some cases, cumbersome instrumentation that can alter the subject’s natural breathing pattern [7,8]. These limitations motivate the investigation of alternative sensing approaches that are more compatible with wearable and ambulatory use.

Bioimpedance analysis has emerged as a versatile and non-invasive methodology for the assessment of several physiological conditions. By measuring the electrical response of biological tissues to low-amplitude alternating currents, bioimpedance-based techniques can provide information related to body composition, tissue electrical properties, respiratory activity, and cardiovascular dynamics [9,10,11]. In particular, bioimpedance spectroscopy (BIS) extends these capabilities by evaluating tissue behavior over a wide frequency range, enabling the extraction of physiologically meaningful parameters through suitable electrical models such as the Cole–Cole representation [12]. For respiratory applications, thoracic bioimpedance is especially attractive because impedance variations are correlated with changes in lung air volume during the breathing cycle, thus enabling unobtrusive monitoring of respiratory dynamics in wearable formats [13,14].

Within this framework, the SENSIPLUS chip, developed by Sensichips S.r.l. in cooperation with the University of Pisa, represents a promising solution for wearable bioimpedance instrumentation. SENSIPLUS is a compact and highly programmable sensor interface that integrates precision low-noise electrical measurement capabilities, including impedance spectroscopy up to 2.5 MHz, while maintaining very low power consumption and a reduced footprint [15,16]. Previous studies have already highlighted its potential in biomedical impedance measurements, showing good agreement with reference instrumentation, satisfactory repeatability, and suitability for integration into portable and IoMT-oriented platforms [17].

This work further explores the use of the SENSIPLUS platform for biomedical monitoring, with particular emphasis on its application to wearable bioimpedance measurements and its potential integration into advanced non-invasive diagnostic systems. By leveraging the flexibility of the SENSIPLUS architecture, the proposed approach aims to contribute to the development of compact, reliable, and scalable tools for continuous health monitoring in both laboratory and real-world settings. Specifically, the paper presents a systematic experimental validation of the platform at three complementary levels: (i) electrical characterisation against tissue-equivalent Debye circuits with three-point bilinear calibration, electrode–skin influence modelling, and repeatability analysis; (ii) multi-frequency in vivo BIS with Cole–Cole model fitting and hook-effect correction, validated against the Agilent E4980A precision LCR meter [18,19]; (iii) single-frequency impedance plethysmography for respiratory monitoring, validated against a calibrated spirometer [20].

The SENSIPLUS chip was originally introduced as a versatile single-chip sensor interface for heterogeneous sensing applications, including vector impedance, voltage, and current measurements over a wide frequency range [15]. Previous works mainly demonstrated its use as a general-purpose EIS-enabled sensing platform [21], for example, on external reference components and in non-biological sensing scenarios such as water-quality assessment [22,23], battery characterization [24], and chemical/gas-sensing applications [25].

In this context, the novelty of the present work is not the SENSIPLUS chip itself, but the systematic investigation of its feasibility for biological bioimpedance measurements through a comprehensive experimental validation protocol. Specifically, this study introduces and experimentally assesses: (i) a three-point bilinear calibration procedure applied to tissue-equivalent Debye circuits over the 10kHz–1MHz range; (ii) an electrode–skin equivalent model to quantify the influence of contact-related artifacts; (iii) a hook-effect correction procedure for in vivo BIS data followed by Cole–Cole parameter extraction; and (iv) a respiratory-monitoring validation against a calibrated spirometer, including both amplitude and temporal analyses. By combining these steps, the manuscript provides a complete metrological and proof-of-concept physiological assessment of the SENSIPLUS-based platform under controlled laboratory conditions.

## 2. Materials and Methods

### 2.1. Measurement Platform

**Figure 1:**
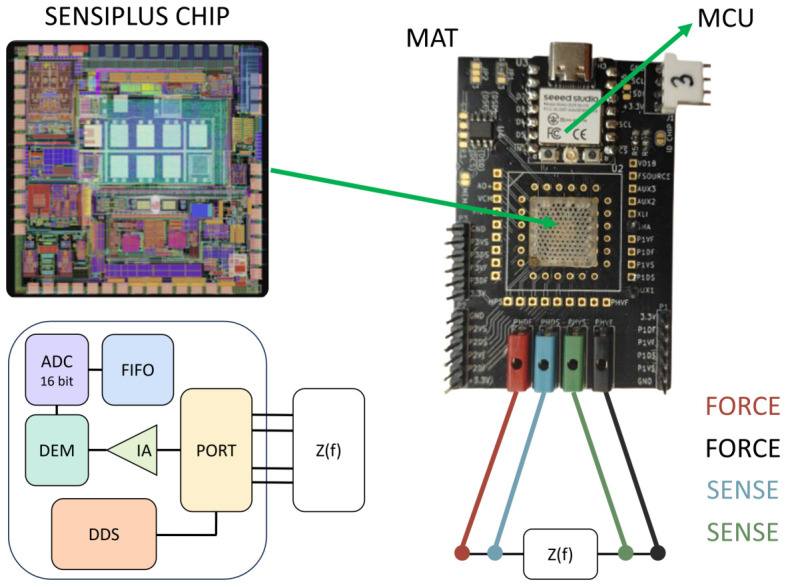
Overview of the proposed measurement platform: the SENSIPLUS chip (top left) is mounted on the MicroAnalytical Tool (MAT) board, which is interfaced to a host computer through a MicroController Unit (MCU). The bottom-left inset shows the simplified block diagram of the on-chip Electrochemical Impedance Spectroscopy (EIS) engine, organized as a lock-in amplifier with stimulus generation and signal acquisition paths.

The experimental setup is based on the SENSIPLUS microchip (Figure 1), a CMOS microelectronic platform designed to integrate multiple heterogeneous sensors within a single device. The chip features a compact footprint of approximately 1.5×1.5 mm^2^ and a power consumption of about 1.5mW during impedance measurements, making it suitable for portable and wearable applications without bulky external instrumentation [15].

At the core of the chip, there is a wideband electrical impedance measurement unit based on a frequency-programmable lock-in amplifier (LIA) architecture, which enables EIS over a wide frequency range. The simplified block diagram of the EIS engine is shown in Figure 1 (bottom-left inset) and can be conceptually divided into two paths. On the stimulus side, the signal generation block, based on a Direct Digital Synthesizer (DDS) and a current driver, produces a programmable sinusoidal excitation that is injected into the unknown impedance Z(f) through the FORCE terminals. On the acquisition side, the voltage developed across the SENSE terminals is first conditioned by a low-noise instrumentation amplifier (IA) and then processed by an I/Q demodulator followed by a low-pass filter, which coherently extracts the in-phase and quadrature components of the response. These are subsequently digitised by a 16-bit Analog-to-Digital Converter (ADC) and processed by a digital back-end comprising a Cascaded Integrator-Comb (CIC) decimation filter and a First-In-First-Out (FIFO) buffer before being transferred to the host interface. By coherent demodulation, the system reconstructs both the real (resistance, R) and imaginary (reactance, X) components of the complex impedance, operating as an integrated precision LCR meter.

In the proposed measurement setup (Figure 1), the SENSIPLUS chip is mounted on the MicroAnalytical Tool (MAT), a Printed Circuit Board (PCB)-based platform that provides the external connectors, power distribution, and digital communication interface required for four-terminal impedance measurements, with dedicated FORCE terminals for current injection and SENSE terminals for voltage sensing. The MAT communicates with an MCU based on the ESP32-C3 architecture through the proprietary SENSIBUS digital interface that, on the MCU side, was implemented in the firmware. The MCU is connected to a host computer via USB, which also provides the system power supply: the MCU operates at 3.3V, derived from the USB regulated 5V rail. This USB-powered configuration was adopted only for supervised benchtop laboratory validation.

In the prototype configuration described in this work, the MAT/SENSIPLUS platform does not include medical-grade galvanic isolation between the host computer/USB power supply and the patient-connected FORCE/SENSE terminals. All critical analog functions, including signal generation, current injection, voltage sensing, coherent demodulation, and impedance reconstruction, are performed by the SENSIPLUS chip. The MCU is used only to translate the commands received from the host computer through the USB connection into the proprietary SENSIBUS digital interface. Measurement configuration and data acquisition are handled by SLM Studio (version 1.3.2) a dedicated software developed by Sensichips s.r.l., which allows the user to set parameters such as excitation frequency, sinusoidal stimulus amplitude, and electrode configuration. Acquired data are exported in CSV format and subsequently processed using custom MATLAB (version 2025a) scripts for signal analysis, calibration, and post-processing.

To accurately characterize the unknown impedance Z(f), the four-terminal (4T) sensing method was employed throughout all measurements. This technique minimizes the contribution of parasitic contact and lead impedances, which would otherwise introduce systematic errors in two-terminal configurations, particularly at low impedance values or high excitation frequencies [26,27]. In this configuration, two electrodes connected to the MAT’s FORCE terminals (red and black connectors in Figure 1) inject an alternating sinusoidal excitation current generated by the SENSIPLUS chip into the unknown impedance Z(f). Simultaneously, two electrodes connected to the SENSE terminals (blue and green connectors in Figure 1) measure the resulting voltage drop directly across the region of interest.

### 2.2. Calibration Procedure

A calibration of the measurement platform was performed to ensure accurate impedance measurements, using three reference loads with known impedance values [28]. This method compensates for systematic errors in the measured impedance by employing a correction function ($Z_{corr}$), which is expressed as follows:

(1) $$Z_{corr}=\frac{Z^{m}-\beta }{-\gamma Z^{m}+\alpha }$$

where $Z^{m}$ is the measured impedance and α, β, and γ are calibration coefficients. Since both calibration and impedance measurements are performed in the frequency domain, all the impedances appearing in Equations (1) and (2) are frequency-dependent quantities. Accordingly, the calibration coefficients are also frequency-dependent and are estimated independently at each investigated frequency point, rather than being treated as global constants over the whole frequency range.

These coefficients are obtained by solving a system of three equations derived from the known reference impedance ($Z_{1},Z_{2},Z_{3}$) and their corresponding measured values ($Z_{1}^{m},Z_{2}^{m},Z_{3}^{m}$):

(2) $$\left\{\begin{array}{c} Z_{1}\alpha +\beta -Z_{1}Z_{1}^{m}\gamma =Z_{1}^{m}\\ Z_{2}\alpha +\beta -Z_{2}Z_{2}^{m}\gamma =Z_{2}^{m}\\ Z_{3}\alpha +\beta -Z_{3}Z_{3}^{m}\gamma =Z_{3}^{m} \end{array}\right.$$

**Figure 2:**
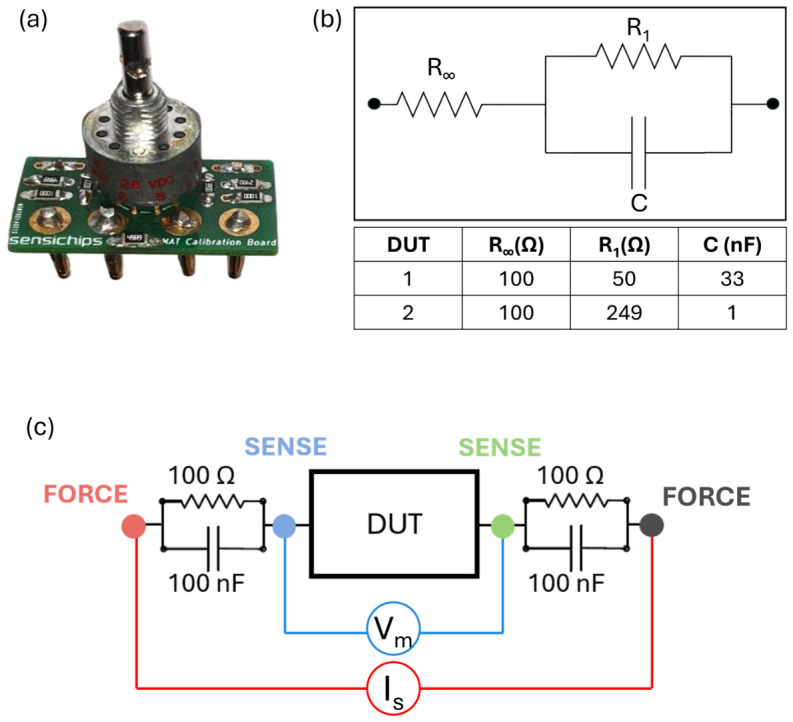
(**a**) Custom test fixture used for calibration purposes. (**b**) The Debye model used for the Devices Under Test (DUT) and the table of values of electrical elements forming tissue equivalent circuits according to the Debye model. (**c**) Schematic of the DUT measurement circuit with the insertion of a parallel RC between Force and Sense, schematizing electrode behavior.

For each frequency point, the three reference impedances $Z_{1}$, $Z_{2}$, and $Z_{3}$, together with the corresponding raw MAT readings $Z_{1}^{m}$, $Z_{2}^{m}$, and $Z_{3}^{m}$, were used to solve Equation (2). The resulting coefficients α, β, and γ were then applied to correct the complex impedance measured at the same frequency using Equation (1). A custom test fixture consisting of a dedicated PCB was designed and fabricated for both system calibration and electrical validation. As illustrated in Figure 2a, the board is equipped with a 5-position rotary switch that enables the user to select between calibration standards and validation circuits. The first three positions connect to high-precision resistors used as reference standards $Z_{1}$, $Z_{2}$, and $Z_{3}$, with nominal values of 50Ω, 220Ω, and 390Ω, respectively. All calibration resistors had a tolerance of ±1%. Before calibration, the actual complex impedance of each standard was measured with the Agilent E4980A LCR meter over the full 10kHz–1MHz frequency range. These measured complex values, rather than the nominal resistance values alone, were used as the reference impedances in the calibration procedure. The remaining two positions connect to tissue-equivalent circuits (DUT1 and DUT2) for electrical validation. The accuracy of the proposed measurement platform was assessed on electronic circuits specifically designed to emulate the frequency-dependent electrical behavior of biological tissues [12,29]. The general framework to describe tissue impedance is based on the Cole–Cole model, expressed as [18]:

(3) $$Z=R_{\infty }+\frac{R_{1}}{1+{\left(j\omega \tau \right)}^{\left(1-\alpha \right)}}$$

where $R_{\infty }$ is the resistance at infinite frequency, $R_{1}$ is the difference between the resistance at zero frequency ($R_{0}$) and $R_{\infty }$, *j* is the imaginary unit, ω represents the angular frequency, τ is the time constant of the circuit (equal to $R_{1}C$), and α (∈[0,1]) is the Cole–Cole distribution factor.

The tissue-equivalent circuits implemented in the test fixture (DUT1 and DUT2) are based on the Debye model (α = 0), which consists of a series resistance $R_{\infty }$ in series with a parallel $R_{1}C$, as shown in Figure 2b. This topology is a special case of the Cole–Cole model with ideal, single-time-constant relaxation behavior. While real biological tissues exhibit a distributed relaxation (α > 0), the Debye model provides a well-defined and reproducible electrical reference for platform validation purposes, as its impedance can be computed analytically with high precision from the nominal component values [30].

The exact impedance of the test fixture was characterized using a precision LCR meter (Agilent Technologies, Santa Clara, CA, USA, model E4980A). According to the manufacturer specifications, the Agilent E4980A provides an impedance accuracy of ±0.3% under the specified operating conditions. Therefore, the residual deviations between the calibrated MAT platform and the LCR meter were interpreted by considering the LCR meter accuracy as the reference-instrument contribution to the measurement uncertainty. Subsequently, the same measurements were performed using the MAT platform to obtain the uncalibrated readings. To guarantee accurate measurements, the four-terminal method is used by the presence of dedicated connectors on the custom test fixture. All tests with the MAT platform were conducted by injecting a sinusoidal current with a peak-to-peak amplitude of approximately 0.65mA across the frequency range of 10kHz to 1MHz.

Once the reference loads ($Z_{1}$, $Z_{2}$, and $Z_{3}$) have been measured using both the LCR meter (Agilent E4980A) and the MAT, a custom MATLAB script is used to solve Equation (2), yielding the calibration parameters α, β, and γ. At this point, all subsequent measurements performed with the MAT will be corrected using the correction formula (1). This allows for the compensation of all systematic errors in the measurement system, thereby improving the overall measurement accuracy.

Although the calibration standards were nominally resistive, their measured impedance was treated as a complex quantity. Therefore, both the residual reactance of the standards and the phase response of the measurement setup were included in the calibration. In Equation (2), $Z_{i}$ and $Z_{i}^{m}$ are complex quantities, and the correction was applied directly to the measured complex impedance $Z^{m}=R^{m}+jX^{m}$. The corrected impedance was then separated into resistance and reactance components as $Z_{corr}=R_{corr}+jX_{corr}$.

For validation purposes, the resistance (*R*) and reactance (*X*) of the tissue-equivalent circuits (DUT1 and DUT2) measured by the calibrated SENSIPLUS platform were directly compared against those obtained with the reference LCR meter.

#### Skin-Electrode Contact Model

An additional investigation was conducted to evaluate the contribution of the electrode–skin interface to the overall measured impedance. Although the four-terminal method substantially mitigates the influence of contact resistance, the electrode–skin interface can still introduce frequency-dependent artifacts, particularly at lower frequencies where the electrode impedance magnitude is non-negligible [31]. To model this effect, the electrode–skin interface was represented by a parallel RC circuit connected between the FORCE and SENSE measurement paths, as illustrated in Figure 2c. The component values (R = 100 Ω, C = 100 nF) were chosen to be representative of typical electrode–skin contact impedance at bioimpedance measurement frequencies [32]. Measurements of DUT1 and DUT2 were repeated with the electrode equivalent network inserted, and the results were compared against those without the electrode model and against the LCR meter reference.

### 2.3. Physiological Measurements

Once the electrical accuracy of the measurement platform was verified using equivalent circuits, the system was further evaluated in two physiological measurement scenarios: bioelectrical impedance spectroscopy (BIS) for tissue characterization and single-frequency impedance plethysmography for respiratory monitoring. These experiments aimed to demonstrate the versatility of the SENSIPLUS-based platform and to quantitatively assess its performance against established reference instruments.

All measurements were performed under standard indoor laboratory conditions. The electrical validation tests, the electrode-equivalent circuit measurements, and the physiological acquisitions were carried out in the same laboratory environment. For each comparison, the acquisitions performed with the MAT platform and with the reference instrumentation were conducted within short time intervals to reduce possible environmental variations between measurements. Ambient temperature and humidity were not actively controlled or continuously logged during the experiments [33]. During the physiological measurements performed with the MAT/SENSIPLUS platform, the host computer was a laptop operated on battery power and disconnected from the AC mains. This precaution was adopted to avoid possible leakage-current paths from the AC mains to the subject-connected measurement setup during the experiments.

#### 2.3.1. Bioelectrical Impedance Spectroscopy

**Figure 3:**
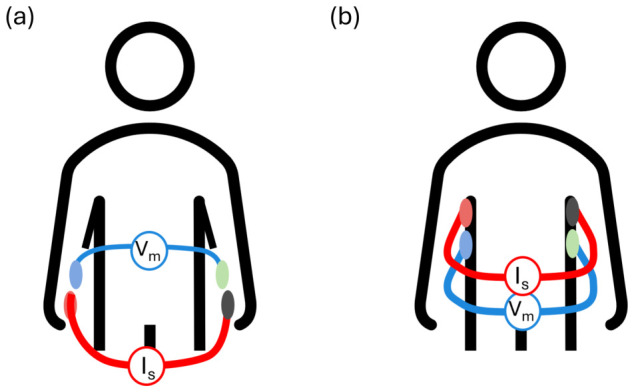
Tetrapolar electrode configurations adopted for the physiological measurements: (**a**) Bioelectrical Impedance Spectroscopy (BIS) setup, with current injection electrodes ($I_{s}$) placed on the wrist and voltage sensing electrodes ($V_{m}$) on the forearm; (**b**) Impedance Plethysmography (IP), with four adhesive electrodes placed symmetrically on the subject’s chest.

Bioimpedance spectroscopy measurements were performed on a healthy volunteer subject to evaluate the in vivo tissue characterization capability of the SENSIPLUS platform. A standard tetrapolar electrode configuration was adopted (Figure 3a): two adhesive electrodes (current injection) were placed on the wrist, while two other adhesive electrodes (voltage sensing) were positioned on the forearm at a mutual separation of approximately 3 cm. The measurements were performed using disposable pre-gelled Ag/AgCl adhesive electrodes, with square geometry and an active contact area of approximately 9 cm^2^. Before electrode placement, the skin was cleaned with alcohol wipes and allowed to dry. The BIS measurements were performed with the subject in a seated position. The MAT was configured to inject a sinusoidal current with a peak-to-peak amplitude of approximately 0.65mA across the frequency range of 10kHz to 1MHz, corresponding to an RMS current of approximately 0.23mA for a sinusoidal waveform. This is well below the reference current limits discussed for patient auxiliary currents in medical instrumentation which, according to [34], must not exceed the 1 mA RMS level at the operating frequency of 10 kHz.

The acquired spectra were fitted to the Cole–Cole model (Equation (3)) using a nonlinear least-squares optimization in MATLAB, yielding the four Cole–Cole parameters ($R_{0}$, $R_{\infty }$, τ, and α). These parameters are widely used in bioimpedance spectroscopy to characterize tissue composition and physiological properties such as intra- and extracellular fluid volumes [10,29]. The reference instrument used for comparison was the precision LCR meter (Agilent E4980A).

#### 2.3.2. Impedance Plethysmography

In addition to impedance spectroscopy measurements, the SENSIPLUS platform was also evaluated for real-time respiratory monitoring using single-frequency impedance plethysmography. This technique is based on the measurement of thoracic bioimpedance variations caused by changes in lung volume during the breathing cycle. During inspiration, expansion of the lungs increases the air volume inside the thorax, which leads to an increase in electrical impedance due to the low conductivity of air. Instead, during expiration, the lung volume decreases, resulting in a reduction in thoracic impedance [35,36]. Measurements were acquired in a tetrapolar configuration using four adhesive electrodes placed symmetrically on the subject’s thorax at the level of the upper ribs. The respiratory plethysmography measurements were performed with the subject in a seated position. The same type of disposable pre-gelled Ag/AgCl adhesive electrodes used for the BIS measurements were adopted for the respiratory plethysmography experiments. Before electrode placement, the skin was prepared using the same procedure described above. A sinusoidal excitation current with a peak-to-peak amplitude of approximately 0.65mA was injected at a fixed frequency of 10kHz (Figure 3b), corresponding to an RMS current of approximately 0.23mA for a sinusoidal waveform.

The measurement protocol consisted of a period of 30 s of normal breathing, followed by 20 s of deep breathing. This cycle was repeated ten times every ten minutes.

The bioimpedance and spirometric signals were acquired with sampling rates of $f_{s,BIOZ}$ = 5 Hz and $f_{s,SPIRO}$ = 10 Hz, respectively. The acquired signals were processed offline using custom MATLAB scripts. The two acquisition streams were synchronized using the common acquisition start time and then visually checked by comparing the temporal position of the main respiratory peaks. The raw bioimpedance signal was first cleaned by removing NaN values and duplicate timestamps, and then filtered with a first-order low-pass Butterworth filter with cut-off frequency $f_{c}=0.7$ Hz, implemented using the MATLAB filtfilt function. This cut-off preserves the physiological respiratory variations while attenuating the cardiac component (∼1–2 Hz) and high-frequency interference. The respiration rate (RR) was estimated from the filtered signal by detecting consecutive peaks. Peak detection was performed using the MATLAB findpeaks function, with a minimum inter-peak distance of 0.3 s, to exclude spuriously close detections, and a minimum prominence equal to 10% of the signal range, to suppress low-amplitude peaks attributable to noise. Breath-by-breath matching between the bioimpedance and spirometer signals was performed by associating each bioimpedance respiratory peak with the nearest spirometer peak. Breaths without a valid matched peak in both signals were excluded from the amplitude and temporal analyses. Overall, 224 breaths were analyzed, including 175 during normal breathing and 49 during deep breathing. The RR was then computed from the set of inter-peak intervals $\left(\Delta t_{k}\right)$ as:

(4) $$RR=\frac{60}{\mathrm{median}(\Delta t_{k})}\;\left[\mathrm{breaths}/\min\right],$$

where $\Delta t_{k}$ is the time interval, expressed in seconds, between two consecutive detected respiratory peaks. The use of the median rather than the mean makes the estimator robust to occasional outlier intervals caused by motion artifacts or missed peaks.

Two complementary analyses were then performed. First, an amplitude analysis was conducted by extracting the peak-to-peak impedance amplitude of each respiratory cycle from both the bioimpedance signal and a calibrated spirometer (used as gold-standard reference instrument), and comparing them via linear regression and Bland–Altman analysis to quantify the correspondence between impedance variations and tidal volume [37]. A temporal analysis was performed by deriving the RR from the maximum peak intervals of the processed impedance waveform and comparing it against the RR derived from the spirometer.

## 3. Results and Discussion

### 3.1. Equivalent Circuit Validation

**Figure 4:**
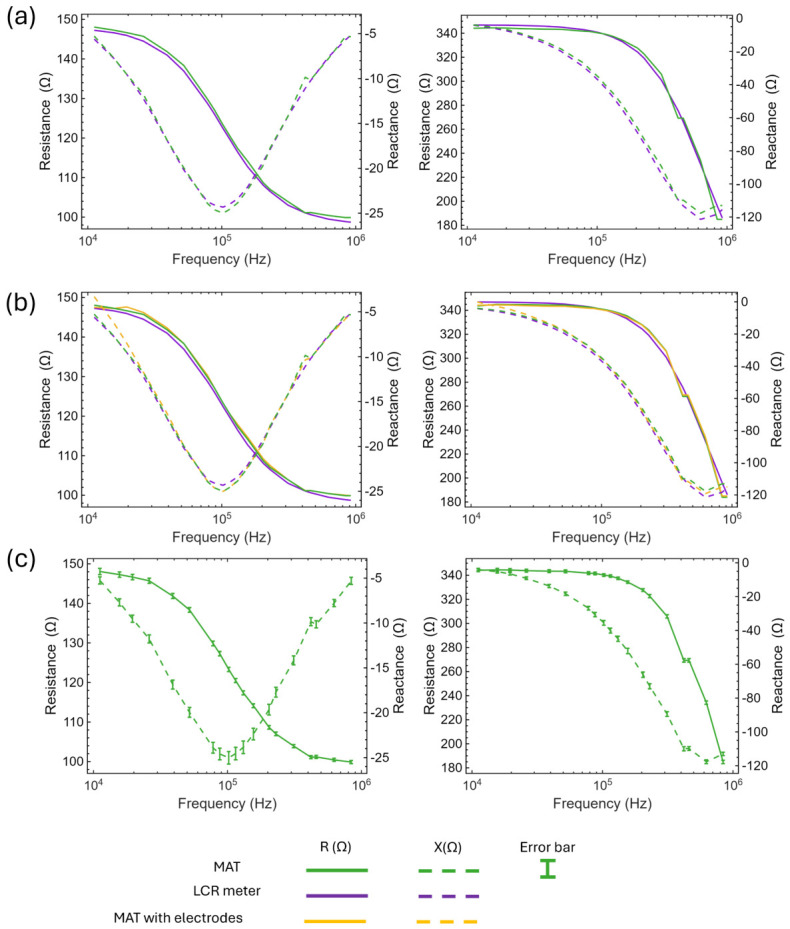
(**a**) Resistance (left y-axis, solid line) and reactance (right y-axis, dashed line) obtained by comparing measurements performed with the MAT platform (green) and the reference LCR meter (purple) for both equivalent circuits (DUT1 and DUT2). (**b**) Resistance (left y-axis, solid line) and reactance (right y-axis, dashed line) of DUT1 and DUT2 measured with the MAT platform without the electrode-equivalent network (green), with the electrode-equivalent network inserted (yellow), and with the reference LCR meter (purple). (**c**) Mean resistance (left y-axis, solid line) and mean reactance (right y-axis, dashed line) of ten repeated measurements of DUT1 and DUT2 with error bars representing one standard deviation.

Figure 4a shows the impedance spectra of the two tissue-equivalent circuits (DUT1 and DUT2) measured with the calibrated MAT platform (green) and the reference LCR meter (purple). Solid lines represent the resistance component *R*, while dashed lines represent the reactance component *X*, in the frequency range from 10kHz to 1MHz. A strong agreement between the two measurement systems can be observed across the entire investigated frequency range for both DUTs. For the resistance component, the maximum relative error between the MAT and the LCR meter was approximately 1.2% at 156kHz for DUT1 and 5.7% at 840kHz for DUT2. For the reactance component, the maximum absolute deviation was 1.24Ω at 420kHz for DUT1 and 5.27Ω at 840kHz for DUT2.

The results confirm the effectiveness of the three-reference-load calibration procedure in correcting systematic errors, providing an impedance measurement accuracy comparable to that of a precision LCR meter. The larger errors observed for DUT2 are attributable to its higher nominal impedance magnitude and to the fact that the maximum error occurs at 840kHz, near the upper limit of the measurement frequency range, where the accuracy of the lock-in amplifier architecture is intrinsically lower due to the decreasing signal-to-noise ratio and the increasing influence of parasitic capacitance within the chip and the PCB interconnects.

#### 3.1.1. Skin-Electrode Contact Analysis

Figure 4b presents the impedance spectra of DUT1 and DUT2 obtained under three different measurement conditions: the reference measurement with the LCR meter (purple), the MAT measurement without the electrode-equivalent network (green), and the MAT measurement with the electrode-equivalent network inserted between the FORCE and SENSE paths (yellow). Solid lines represent the resistance component *R*, while dashed lines represent the reactance component *X*.

For DUT1, the introduction of the electrode-equivalent circuit increases the maximum relative error on the resistance component from 1.2% to 1.71% at 156kHz. The effect is higher on the reactance component, where the maximum absolute error increases from 1.24Ω at 420 kHz to 2.29Ω at 11 kHz, showing that the electrode artifact is stronger at lower frequencies.

For DUT2, a similar trend was observed. The maximum relative error on the resistance increases from 5.71% (without electrode equivalent circuits) to 6.22% (with electrode equivalent circuits) at 840kHz. For the reactance component, the maximum absolute error changes from 5.27Ω at 840 kHz to 4.03Ω at 11 kHz. The introduction of the electrode network shifts the frequency at which the maximum absolute error on the reactance occurs.

These results are consistent with the capacitive nature of the electrode–skin interface [38]: at low frequencies (e.g., 11kHz), the impedance of the electrode RC network is relatively large and its contribution to the measured reactance is non-negligible (the error on *X* increases from 0.33Ω to 2.28Ω for DUT1, and from 0.13Ω to 4.03Ω for DUT2). As the excitation frequency increases, the impedance of the electrode network decreases, and the error on the reactance is correspondingly reduced (from 1.23Ω to 0.74Ω at 420kHz for DUT1, and from 5.73Ω to 3.33Ω at 840kHz for DUT2). The resistance, by contrast, exhibits only a slight overall increase in the error across the spectrum, since the four-terminal configuration effectively suppresses the in-phase contribution of the electrode impedance.

#### 3.1.2. Repeatability Analysis

To assess measurement repeatability, ten consecutive impedance measurements of DUT1 and DUT2 were performed under identical experimental conditions. Figure 4c shows the mean resistance (solid line) and mean reactance (dashed line), with error bars representing the standard deviation across the ten repetitions.

The standard deviation was consistently low across the full frequency range for both circuits. The largest variability was observed at 10kHz for both DUTs. For DUT1, the maximum standard deviation was 0.76Ω for the resistance and 0.55Ω for the reactance. For DUT2, the corresponding values were 1.37Ω and 0.97Ω, respectively. The slightly higher dispersion observed at low frequencies is consistent with the reduced signal-to-noise ratio of the lock-in amplifier in this region, where the integration time and the magnitude of the demodulated components are most susceptible to random fluctuations. These results confirm the good repeatability of the SENSIPLUS-based platform for impedance spectroscopy.

### 3.2. Bioimpedance Spectroscopy Measurements

Bioimpedance spectroscopy measurements were performed on a volunteer subject to evaluate the capability of the proposed platform to characterize biological tissues in vivo. Measurements were acquired with both the MAT platform and the reference LCR meter using the same tetrapolar electrode configuration in two sequential acquisitions. The electrodes were kept in place throughout the two measurements, and the time interval between the acquisitions was minimized to reduce changes in the electrode–skin interface and in the physiological state of the subject. The reference LCR meter was configured to inject a maximum effective current of 1mA in the 10kHz–1MHz band.

**Figure 5:**
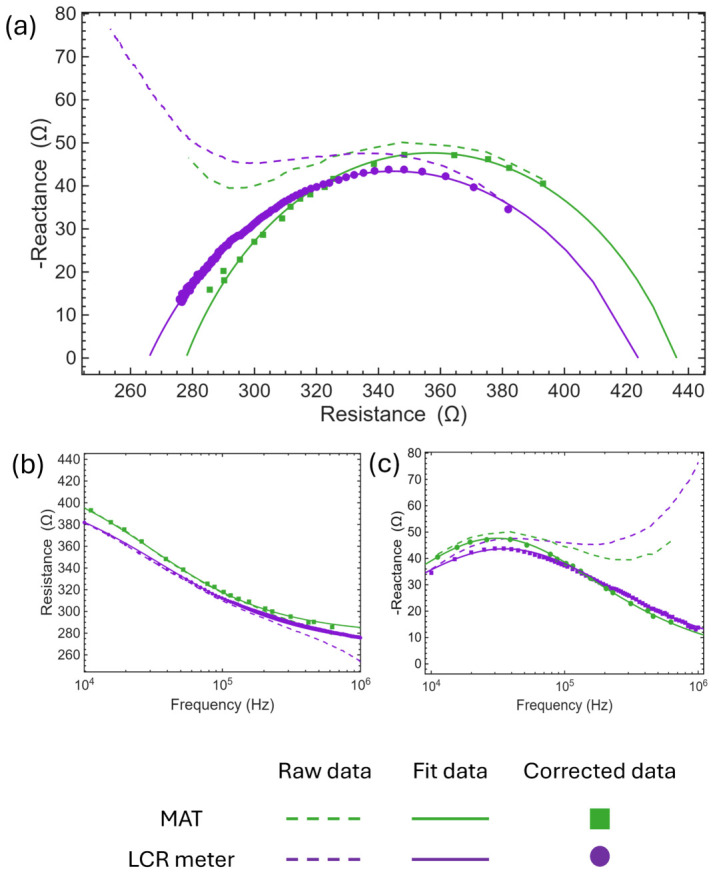
Comparison of (**a**) Cole–Cole (**b**) resistance, and (**c**) reactance measurements collected using MAT before (green dashed line) and after (green squares) correction, and LCR Meter before (purple circle markers) and after (purple line) correction.

Figure 5a shows the Cole–Cole plot of the measured impedance obtained with the tetrapolar electrode configuration of Figure 3a.

The raw values of the bioimpedance acquired with the MAT, $Z_{MAT}$ (green dashed line), and with the LCR meter, $Z_{LCR}$ (purple dashed line), exhibit a clear deviation from the expected semicircular Cole–Cole locus in the high-frequency region (low-resistance branch, between approximately 250Ω and 340Ω). This deviation corresponds to the well-known *hook artifact*, typically attributed to a parasitic capacitance of the measurement setup [19]. The hook effect was compensated by applying the following correction [39]:

(5) $$Z_{hook}=Z_{meas}\cdot \left(\frac{1}{1-j\omega Z_{meas}C_{p}}\right)$$

where $Z_{meas}$ is the impedance measured by the instrument and $C_{p}$ is the equivalent parasitic capacitance. The hook-effect correction was applied separately to the MAT and LCR datasets. For each dataset, $C_{p}$ was estimated together with the Cole–Cole parameters by minimizing the squared residuals between the corrected complex impedance data and the Cole–Cole model over the measured frequency range. The optimization was performed in MATLAB using a nonlinear least-squares routine applied simultaneously to the real and imaginary components of the impedance. The fitted parameter vector was $\theta =[R_{0},R_{\infty },\tau ,\alpha ,C_{p}]$. The bounds used in the optimization were $290\;\Omega \leq R_{0}\leq 470\;\Omega$, $260\;\Omega \leq R_{\infty }\leq 300\;\Omega$, 5μs≤τ≤40ms, 0≤α≤1, and $0\leq C_{p}\leq 500\;\mathrm{pF}$. The initial values were set within the admissible intervals as $R_{0}=380\;\Omega$, $R_{\infty }=280\;\Omega$, τ=5μs, α=0, and $C_{p}=1\;\mathrm{pF}$.

After compensation, the MAT data ($Z_{MAT,\;corrected}$, green circles) recover the expected semicircular trajectory and are well fitted by the Cole–Cole model (green solid line) with parameters $R_{0}=436.20\;\Omega$, $R_{\infty }=277.89\;\Omega$, τ=5.14μs, and α=0.31, with an estimated parasitic capacitance $C_{p}=100\;\mathrm{pF}$. Equivalently, the LCR meter corrected data ($Z_{LCR,\;corrected}$, purple squares) also follow a clear semicircular profile, yielding $R_{0}=423.80\;\Omega$, $R_{\infty }=268.89\;\Omega$, τ=4.51μs, and α=0.36, with $C_{p}=145\;\mathrm{pF}$ (purple solid line). $C_{p}$ should be interpreted as an equivalent fitted parameter representing the overall parasitic contribution of the complete measurement chain. The full set of estimated Cole–Cole parameters for both instruments is summarized in Table 1. To assess the sensitivity of the fitting procedure to the initialization of the parasitic-capacitance correction, the optimization of $C_{p}$ was repeated using different initial values distributed across the admissible range, namely 1 pF, 100 pF, 200 pF, 300 pF, 400 pF, and 500 pF. For each initialization, $C_{p}$ was optimized by minimizing the residual difference between the Cole–Cole model and the hook-corrected impedance data. The optimization converged to the same minimum within numerical tolerance for all tested initializations, indicating that the corrected impedance spectra and the resulting Cole–Cole parameters were not dominated by the initial value assigned to $C_{p}$.

Figure 5b,c show the resistance and reactance spectra as a function of frequency for both instruments. In the resistance plot (Figure 5b), the corrected MAT data (green markers and solid fit line) and the corrected LCR meter data (purple markers and solid fit line) both exhibit the monotonically decreasing dispersive behavior expected for biological tissues.

The two instruments display a relatively small offset, in the order of 10–15Ω, which corresponds to the small variations in R_0_ and R_∞_ in Table 1. This offset can be ascribed to a combination of factors, including the residual calibration uncertainty of the MAT platform, the different excitation current amplitudes used by the two instruments (0.65mA for the MAT and 1mA for the LCR meter), and the micro-movements of the subject between the two sequential acquisitions.

In the reactance spectrum (Figure 5c), both plots exhibit the characteristic bell-shaped curve, with the reactance reaching a peak value of approximately −47Ω at 31kHz for the MAT and −44Ω at 35kHz for the LCR meter. These peak frequencies are in excellent agreement with the characteristic frequencies derived from the Cole–Cole fit ($f_{Cole-Cole}=1/\left(2\pi \tau \right)\approx 31\;\mathrm{kHz}$ for the MAT and ≈35kHz for the LCR meter). After the hook-effect correction, both datasets align well with their respective Cole–Cole fits, demonstrating the effectiveness of the compensation procedure and confirming the capability of the SENSIPLUS-based platform to reliably characterize the dispersive electrical properties of biological tissues over a wide frequency range.

### 3.3. Experimental Validation of Impedance Plethysmography

**Figure 6:**
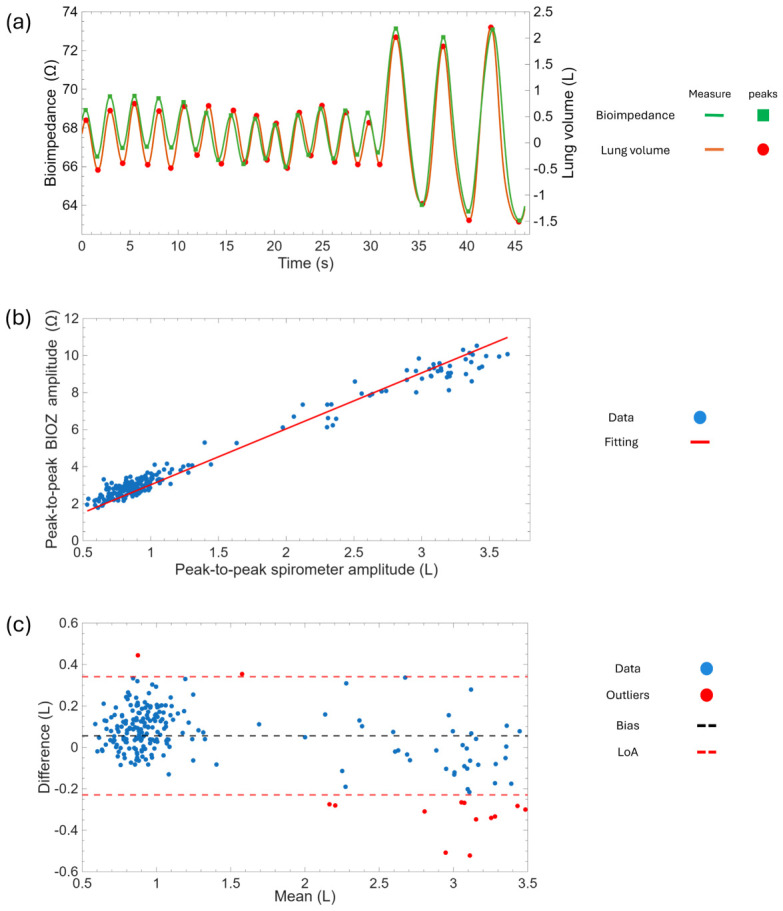
Single-frequency impedance plethysmography results: (**a**) temporal comparison between the thoracic bioimpedance signal (BIOZ, green, left axis) and the reference spirometer signal (orange, right axis), with detected maxima and minima marked for both signals; (**b**) linear regression between peak-to-peak bioimpedance amplitude and peak-to-peak spirometric amplitude (one point per respiratory cycle); (**c**) Bland–Altman plot of the breath-by-breath agreement between bioimpedance-derived and spirometer-derived respiratory amplitudes.

Figure 6a shows the temporal comparison between the thoracic bioimpedance signal and the reference spirometer signal during a representative impedance plethysmography session. The thoracic bioimpedance waveform (green, left y-axis) and the corresponding spirometric volume signal (orange, right y-axis) are plotted as a function of time, together with the detected maxima and minima of both signals (green squares for the BIOZ signal and red circles for the spirometer). A strong temporal correspondence between the two signals is observed over the entire measurement window, and the two phases of the measurement protocol can be clearly identified: during the first ∼30s, the low-amplitude oscillations correspond to normal breathing; after 30s, deep-breathing cycles produce larger-amplitude variations that are consistently reproduced in both signals.

#### 3.3.1. Amplitude Correlation

To quantify the relationship between bioimpedance-derived and spirometer-derived respiratory volumes, the peak-to-peak amplitude of each respiratory cycle was extracted from both signals and compared via linear regression. Figure 6b shows the resulting scatter plot, with the peak-to-peak spirometric amplitude on the x-axis and the corresponding peak-to-peak bioimpedance amplitude (SENSIPLUS platform) on the y-axis. Two well-separated clusters can be identified, corresponding to normal breathing (lower peak-to-peak values) and deep breathing (higher peak-to-peak values). Despite a slight increase in dispersion at higher amplitudes, the linear trend is preserved across the entire measurement range, and the coefficient of determination ($R^{2}=0.97$) confirms a strong linear proportionality between thoracic impedance variation and tidal volume [40]. The overall slope of the regression line, m=3.02Ω/L (for this participant), provides the conversion factor required to express the bioimpedance signal in equivalent volumetric units:

(6) $$V_{BIOZ}=\frac{\Delta Z}{m}$$

where ΔZ is the peak-to-peak bioimpedance variation in a given respiratory cycle, and $V_{BIOZ}$ is the corresponding bioimpedance-derived respiratory amplitude expressed in equivalent volumetric units. Because the pooled dataset comprised two distinct amplitude regimes, the regression analysis was also repeated separately for normal and deep breathing cycles, using the corresponding phases of the experimental protocol. For normal breathing, linear regression constrained to the origin yielded ΔZ=3.21·ΔV, with $R^{2}=0.73$. For deep breathing, the resulting relationship was ΔZ=2.92·ΔV, with $R^{2}=0.74$. Compared with the pooled sensitivity of 3.02Ω/L, the slopes differed by approximately 6.3% for normal breathing and 3.3% for deep breathing. These values are reasonably consistent with the global analysis, supporting the use of the pooled sensitivity as a reference conversion factor for the overall intra-subject impedance-to-volume comparison. To further assess the breath-by-breath agreement between the two measurement methods, a Bland–Altman analysis was performed (Figure 6c). The difference between the bioimpedance-derived and spirometer-derived amplitudes is plotted against their mean value, together with the bias and the limits of agreement (LoA), defined as:

(7) LoA=bias±1.96σ

where σ is the standard deviation of the differences. The majority of the data points fall within the limits of agreement, indicating good consistency between the two methods.

The Bland–Altman analysis showed a small overall bias of approximately 0.05L, with limits of agreement of approximately −0.24L and 0.34L. The number of outliers remains limited (approximately 6.2% of the breaths), while the points associated with normal breathing remain tightly clustered around the mean difference. These results indicate that, when all respiratory cycles are considered together, the bioimpedance-derived volume estimate shows limited average bias with respect to the spirometer reference.

However, the distribution of the points was not uniform across the investigated volume range. The cycles corresponding to normal breathing were clustered at lower mean volumes and showed a narrower dispersion around the bias line, whereas the deep-breathing cycles were associated with larger mean volumes and wider dispersion. Therefore, the Bland–Altman analysis was also repeated separately for normal and deep breathing. For normal breathing, the bias was 0.09L, with limits of agreement from −0.10L to 0.29L. For deep breathing, the bias was −0.08L, with wider limits of agreement from −0.46L to 0.29L. The wider limits of agreement observed during deep breathing confirm the larger dispersion already visible in Figure 6c. This behavior may be attributed to stronger thoracic motion, larger deformation of the electrode–skin interface, residual synchronization uncertainty between the two acquisition chains, and physiological variability during voluntary deep-breathing maneuvers. Overall, these results support the feasibility of intra-subject respiratory-amplitude tracking using the SENSIPLUS-based platform, while also indicating that further validation and signal-processing refinement are required before drawing conclusions on general quantitative accuracy across subjects and breathing conditions.

#### 3.3.2. Temporal Analysis

The temporal accuracy of the SENSIPLUS platform was evaluated by comparing the respiratory rate (RR), expressed in breaths per minute (BPM), derived from the bioimpedance signal with respect to the spirometer reference. The RR was computed by detecting consecutive respiratory cycles and measuring the time interval between two peaks. The results obtained across ten measurement sessions are summarized in Table 2, where the mean respiratory rates during normal and deep breathing are reported for both instruments, together with the corresponding relative errors.

The overall mean RR was 23.39BPM (BIOZ) versus 23.29BPM (spirometer) during normal breathing (mean relative error equal to 1.43%), and 14.25BPM (BIOZ) versus 14.29BPM (spirometer) during deep breathing (mean relative error equal to 1.96%). The overall mean relative errors were below 2% in both breathing regimes, namely 1.43% during normal breathing and 1.96% during deep breathing. However, this value refers to the average error across the ten sessions, not for each individual session. The higher deviations observed in a limited number of sessions, especially during deep breathing, may also be related to the different physiological quantities sensed by the two measurement modalities. While the spirometer directly reflects airflow and lung-volume changes, thoracic bioimpedance is influenced not only by lung air content but also by changes in intrathoracic fluid distribution, blood volume, thoracic deformation, and electrode–skin interface conditions. During voluntary deep-breathing maneuvers, larger thoracic expansion may therefore induce additional bioimpedance variations and small temporal shifts with respect to the spirometric waveform.

These results support the feasibility of respiratory rate and phase tracking with the SENSIPLUS-based platform in the investigated single-subject protocol. However, they should not be interpreted as evidence of clinical accuracy or generalizability across subjects, pathological conditions, or long-term wearable use [41].

## 4. Conclusions

This paper presented a comprehensive experimental validation of a compact bioimpedance measurement platform based on the SENSIPLUS chip, covering electrical characterization, in vivo bioimpedance spectroscopy, and single-frequency impedance plethysmography for respiratory monitoring.

At the electrical validation level, the three-point bilinear calibration procedure reduced the maximum relative resistance error to 1.2% for DUT1 and 5.71% for DUT2, with maximum reactance absolute errors of 1.24 Ω and below 6 Ω, respectively. The Debye model tissue-equivalent circuits provided an analytically exact, reproducible ground truth, enabling rigorous quantification of the residual systematic errors after calibration. The electrode–skin interface analysis confirmed that its contribution is predominantly capacitive and concentrated at frequencies below approximately 16 kHz, consistent with the RC model values adopted. Repeatability was assessed through ten consecutive measurements, yielding maximum standard deviations of 0.76 Ω (R) and 0.55 Ω (X) for DUT1, and 1.37 Ω (R) and 0.97 Ω (X) for DUT2.

For multi-frequency BIS, the platform successfully characterized the in vivo forearm impedance spectrum after hook-effect correction ($C_{p}=0.1$ nF), yielding Cole–Cole parameters ($R_{0}=436.20$ Ω, $R_{\infty }=277.89$ Ω, $\tau =5.14\times 10^{-6}$ s, α=0.31) consistent with published reference values for healthy subjects. These results suggest the potential of the platform for tissue characterization applications; assessment of body composition and fluid status will require dedicated validation on larger, independent cohorts. For single-frequency impedance plethysmography, a strong linear correlation between thoracic bioimpedance variation and spirometric tidal volume was demonstrated ($R^{2}=0.97$, slope m=3.02 Ω/L), with 93.8% of breath-by-breath measurements falling within the Bland–Altman limits of agreement (±0.33 L). RR estimation showed mean relative errors below 2% across the ten sessions, namely 1.43% during normal breathing and 1.96% during deep breathing. These results support the feasibility of using the SENSIPLUS platform for respiratory rate estimation in the investigated single-subject protocol. However, they should not be interpreted as evidence of clinical accuracy or generalizability across subjects, pathological conditions, or long-term wearable use. It should be noted that the physiological measurements presented in this work were performed on a single healthy volunteer and were intended as a proof-of-concept validation of the proposed platform rather than a clinical study. Therefore, the reported physiological results should be interpreted as evidence of the technical feasibility and measurement performance of the system. Future work will therefore focus on validation in larger and independent cohorts, including subjects with different age, sex, body composition, hydration status, and respiratory patterns. Dedicated studies will also be required to assess the accuracy of the platform for body-composition and fluid-status monitoring, as well as its robustness during prolonged wearable acquisitions, where electrode–skin stability, motion artifacts, posture, and environmental conditions may affect the measured bioimpedance. Long-term acquisitions will be performed to specifically evaluate the time-dependent variation in the electrode–skin contact impedance during continuous monitoring, with particular attention to electrode stabilization, skin hydration, perspiration, and motion-related artifacts. Moreover, the translation of the current offline MATLAB signal-processing pipeline into lightweight edge-deployable implementations will be addressed, for example using embedded firmware or portable software frameworks, with a view to providing better support for real-time wearable and IoMT-oriented operation. The reported results demonstrate that the SENSIPLUS-based platform achieves a metrological performance suitable for both multi-frequency spectroscopy and single-frequency physiological monitoring applications, while maintaining the compact footprint (1.5 × 1.5) mm^2^ and low power consumption (1.5 mW) which are widely identified as prerequisites for integration into wearable IoMT devices. It should also be noticed that the compact footprint and low-power figures reported in this work refer to the SENSIPLUS chip itself, which performs all critical analog functions required for impedance measurement. The MAT board was used in this study to simplify laboratory experimentation by providing connectors, power distribution, and digital communication with the host computer. As implemented here, the complete USB-powered MAT-based setup, including the microcontroller, communication interface, and host-computer processing, is not a fully wearable system and has size and power requirements higher than those of the SENSIPLUS chip alone. In the present configuration, the MAT board draws approximately 110mW from the 5V USB power line. Nevertheless, since SENSIPLUS performs the critical analog operations, its integration with a compact low-power microcontroller and optimized embedded processing can be reasonably envisioned as a further step toward a fully wearable implementation.

### Limitations and Safety Considerations

The physiological experiments reported in this work were performed under supervised laboratory conditions and were intended only as a technical proof-of-concept validation. The present MAT/SENSIPLUS implementation should not be considered a medical device or a clinically deployable system in its current configuration. In particular, the USB-powered setup described here does not provide certified medical-grade galvanic isolation or dedicated Means of Patient Protection between the host computer/power supply and the subject-connected electrodes. Therefore, future clinical or wearable implementations will require a dedicated safety-oriented redesign, including galvanic isolation of power and data lines, leakage-current control, and verification of compliance with the applicable medical electrical equipment standards. Moreover, the present prototype should not be used in subjects with known cardiac disease, arrhythmias, or implanted electronic devices such as pacemakers or implantable cardioverter-defibrillators, especially for transthoracic electrode configurations.

## Figures and Tables

**Table 1:** Cole–Cole parameters and parasitic capacitance estimated from the MAT platform and the reference LCR meter on the in vivo bioimpedance spectrum.

Parameter	MAT Platform	LCR Meter	Relative Deviation
$R_{0}$ (Ω)	436.20	423.80	2.93%
$R_{\infty }$ (Ω)	277.89	268.89	3.35%
τ (μs)	5.14	4.51	13.97%
α	0.31	0.36	13.89%
$C_{p}$ (pF)	100	145	–

**Table 2:** Comparison of RR estimated from the spirometer and the bioimpedance (BIOZ) signal for multiple measurement sessions. The relative error is reported for both normal and deep breathing conditions.

Session	Mean RR SPIRO (BPM)		Mean RR BIOZ (BPM)		Rel. Error (%)	
	Normal	Deep	Normal	Deep	Normal	Deep
1	20.79	13.28	21.15	13.51	1.68	1.74
2	25.34	14.44	25.67	14.66	1.29	1.54
3	22.96	13.50	23.09	13.36	0.54	1.01
4	22.64	15.32	22.89	15.50	1.09	1.15
5	22.05	13.68	22.44	13.97	1.76	2.10
6	23.07	13.37	23.41	13.49	1.43	0.93
7	19.21	13.64	19.18	13.78	0.16	1.01
8	25.98	15.26	26.25	14.90	1.06	2.36
9	26.03	15.45	24.84	14.51	4.57	6.09
10	24.78	14.99	24.96	14.74	0.68	1.65
**Overall**	23.29	14.29	23.39	14.25	1.43	1.96

## Data Availability

The data presented in this study are available from the corresponding author upon reasonable request. The data are not publicly available because they are part of an ongoing research project.
